# Dynamic Procalcitonin Trajectories Predict Adverse Outcomes in Acute-on-Chronic Pancreatitis

**DOI:** 10.7759/cureus.113242

**Published:** 2026-07-23

**Authors:** Dibya J Sharma, Bikram Chowdhury, Abhisek Ghosh, Abhinav Nath, Phulen Sarma

**Affiliations:** 1 Internal Medicine, Gastroenterology, Silchar Medical College and Hospital, Silchar, IND; 2 Medicine, Silchar Medical College and Hospital, Silchar, IND; 3 General Medicine, Silchar Medical College and Hospital, Silchar, IND; 4 Pharmacology, All India Institute of Medical Sciences, Guwahati, Guwahati, IND

**Keywords:** acute-on-chronic pancreatitis, biomarker, inflammation, intensive care, mortality, organ failure, procalcitonin, prognosis, systemic

## Abstract

Background and objective

Acute-on-chronic pancreatitis (ACP) is an increasingly recognized clinical entity associated with significant morbidity, prolonged hospitalization, and increased intensive care utilization. Early identification of high-risk patients remains challenging because conventional prognostic scoring systems are often time-consuming, complex, or insufficiently validated in ACP. Procalcitonin (PCT), a rapid inflammatory biomarker, has demonstrated prognostic utility in acute pancreatitis. However, evidence regarding its role in ACP remains limited. This study aimed to evaluate serial serum PCT levels in hospitalized patients with ACP from Northeast India.

Methods

This prospective observational study was conducted over one year at a tertiary care center and included 58 adult patients diagnosed with ACP based on the revised Atlanta classification criteria and imaging evidence of chronic pancreatitis (CP). Serial serum PCT levels were measured at admission and 48 hours later. Clinical outcomes, including mortality, ICU admission, organ dysfunction, local complications, and duration of hospitalization, were analyzed. Receiver operating characteristic (ROC) curve analysis was performed to assess the prognostic accuracy of PCT.

Results

Severe clinical outcomes were common, with mortality observed in 12.1% (n = 7) of patients, ICU admission in 60.3% (n = 35), and local pancreatic complications noted among 82.8% (n = 48) of the cases. Admission PCT demonstrated poor prognostic performance for mortality prediction (area under the curve (AUC): 0.555), with a sensitivity of 46.9% at a cutoff of 3.46 ng/mL. In contrast, 48-hour PCT showed excellent predictive accuracy, yielding an AUC of 0.992. A threshold value of 4.04 ng/mL achieved 100% sensitivity and 100% negative predictive value (NPV) for mortality prediction. Elevated PCT levels were significantly associated with ICU admission (p = 0.034), organ dysfunction (p = 0.021), local complications (p = 0.041), and prolonged hospitalization (r > 0.95).

Conclusions

Serial serum PCT assessment, particularly at 48 hours, is a reliable prognostic biomarker in ACP and may facilitate early risk stratification, optimized critical care allocation, and individualized clinical management.

## Introduction

Acute pancreatitis (AP) carries a sizable disease burden, with about a quarter of patients progressing to severe sepsis and requiring intensive care [1,2]. Up to a quarter of patients also experience recurrent episodes, while more than 10% may progress to chronic pancreatitis (CP), and approximately 10% to 35% experience recurrent acute exacerbations [2,3]. Acute-on-chronic pancreatitis (ACP), defined as an acute exacerbation of the inflammatory process in a patient with established CP, has gained recent interest due to its high rate of ICU admissions, prolonged hospital stays, and increased risk of local complications [4]. While clinical consensus on the distinctiveness of ACP is evolving, it is widely recognized as the most appropriate term to describe acute inflammatory changes in patients with CP [4]. ACP is distinguished from isolated AP by unique characteristics, differing biomarker profiles, and morphological findings on imaging [5,6,7]. It is also distinct from recurrent acute pancreatitis (RAP), which typically occurs before structural CP has been established [4].

Despite this consensus, prognostic prediction in ACP remains difficult, with diagnosis still relying heavily on cross-sectional imaging [8,9]. There is an urgent clinical need for a rapid, simple tool to predict the need for early ICU admissions and effectively reduce mortality [8,10]. Current prognostic tools lack the capability for timely risk stratification. The Ranson criteria are delayed by a 48-hour assessment window, while the Acute Physiology and Chronic Health Evaluation II (APACHE II) score is often too complex for emergency settings [11]. The Bedside Index for Severity in Acute Pancreatitis (BISAP) score and C-reactive protein (CRP) are frequently outperformed by biochemical markers with faster kinetics, such as procalcitonin (PCT), which can identify high-risk patients within the first 12-24 hours [12,13,14,15].

PCT, a 116-amino acid precursor of calcitonin, is a highly sensitive biomarker that reflects systemic inflammatory activation [16,17]. During pancreatic injury, PCT is secreted by multiple organs in response to tissue damage and pathogen-associated signals [1,17]. Landmark studies have established its role in predicting infected necrosis (cutoff of 11.8 ng/mL) and organ failure within 12 hours of admission [12,18]. Meta-analyses suggest that PCT levels above 0.5 ng/mL reliably identify severe pancreatitis [19]. However, specific evidence for ACP remains scarce, as most protocols are adapted from AP guidelines [4]. We therefore conducted this prospective study to evaluate serial serum PCT levels in hospitalized patients with ACP from Northeast India.

## Materials and methods

Study design

This was a prospective observational study conducted in a hospital-based setting over a period of one year. Patients presenting with ACP were enrolled at admission and followed throughout their hospital stay. The design allowed for the systematic documentation of clinical, biochemical, and imaging parameters without altering routine management, thereby ensuring that the findings reflected real-world practice.

Study setting

The study took place at a tertiary care referral hospital in Assam, India, which serves as a major center for advanced gastroenterology and hepatology. Assam and the North-Eastern region carry a considerable burden of pancreatitis, with alcohol-related and idiopathic cases being particularly common. As a referral center, the hospital receives complex cases from surrounding districts, providing a diverse patient population that is suitable for evaluating ACP.

Patient recruitment

Patients were recruited consecutively to minimize selection bias. All individuals presenting with suspected ACP during the study period underwent eligibility screening, which included clinical evaluation, laboratory testing, and imaging confirmation. Written informed consent was obtained from each participant before enrollment. Patients meeting the inclusion criteria were followed prospectively until discharge or death. A flowchart was prepared to illustrate the recruitment process, including the number of patients screened, excluded, and analyzed, thereby ensuring transparency in patient selection.

ACP diagnostic criteria

AP was defined according to the Revised Atlanta criteria, requiring at least two of the following: characteristic abdominal pain, serum amylase or lipase activity greater than three times the upper limit of normal, and imaging findings consistent with AP. CP was diagnosed based on imaging evidence of pancreatic calcifications, ductal irregularities, or parenchymal atrophy. Operationally, ACP was defined as AP fulfilling the Revised Atlanta criteria in patients with imaging evidence of established CP. This definition ensured accurate classification of patients with established chronic pancreatic damage experiencing acute inflammatory exacerbations. The distinction was critical, as ACP differs from isolated AP in its clinical course, severity, and outcomes. Applying standardized diagnostic criteria reduced the risk of misclassification and enhanced comparability with existing literature.

Inclusion and exclusion criteria

Adults aged 18 years and above presenting with clinical features of AP and imaging evidence of CP were eligible for inclusion. Patients with alternative diagnoses such as gallbladder disease, malignancy, or non-pancreatic abdominal pathology were excluded. Those with incomplete imaging, refusal of consent, or prior enrollment in similar studies were also excluded. These criteria ensured a homogeneous study population and minimized potential confounding factors.

Clinical and laboratory assessment

Baseline demographic data, including age, sex, and socioeconomic background, were recorded. Clinical history focused on comorbidities such as diabetes, hypertension, and cardiovascular disease, as well as lifestyle factors, including alcohol consumption and smoking. Laboratory investigations included serum amylase and lipase, liver function tests, renal function tests, and complete blood counts. Imaging studies, primarily contrast-enhanced CT (CECT), were performed to assess pancreatic morphology, necrosis, and complications. These parameters provided a comprehensive clinical and biochemical profile for each patient, enabling correlation with PCT levels and outcomes.

Procalcitonin measurement

Serum PCT levels were measured using a quantitative immunoassay based on electrochemiluminescence (Roche Diagnostics, Indianapolis, IN). Samples were collected at admission and after 48 hours to capture dynamic changes. Calibration was performed according to the manufacturer’s instructions, and internal quality controls were run with each batch. The laboratory reference range for healthy individuals was < 0.05 ng/mL. All assays were conducted in the hospital’s central laboratory, ensuring standardized procedures and reproducibility.

Outcomes

The primary outcome was a composite endpoint comprising in-hospital mortality, ICU admission, and persistent organ failure lasting for more than 48 hours. Secondary outcomes included local complications such as pancreatic necrosis and pseudocyst formation, prolonged hospital stay beyond 14 days, and the need for surgical or endoscopic intervention. These outcomes were chosen to reflect both systemic and local disease severity, providing a comprehensive assessment of ACP progression.

Statistical analysis

**Figure 1:**
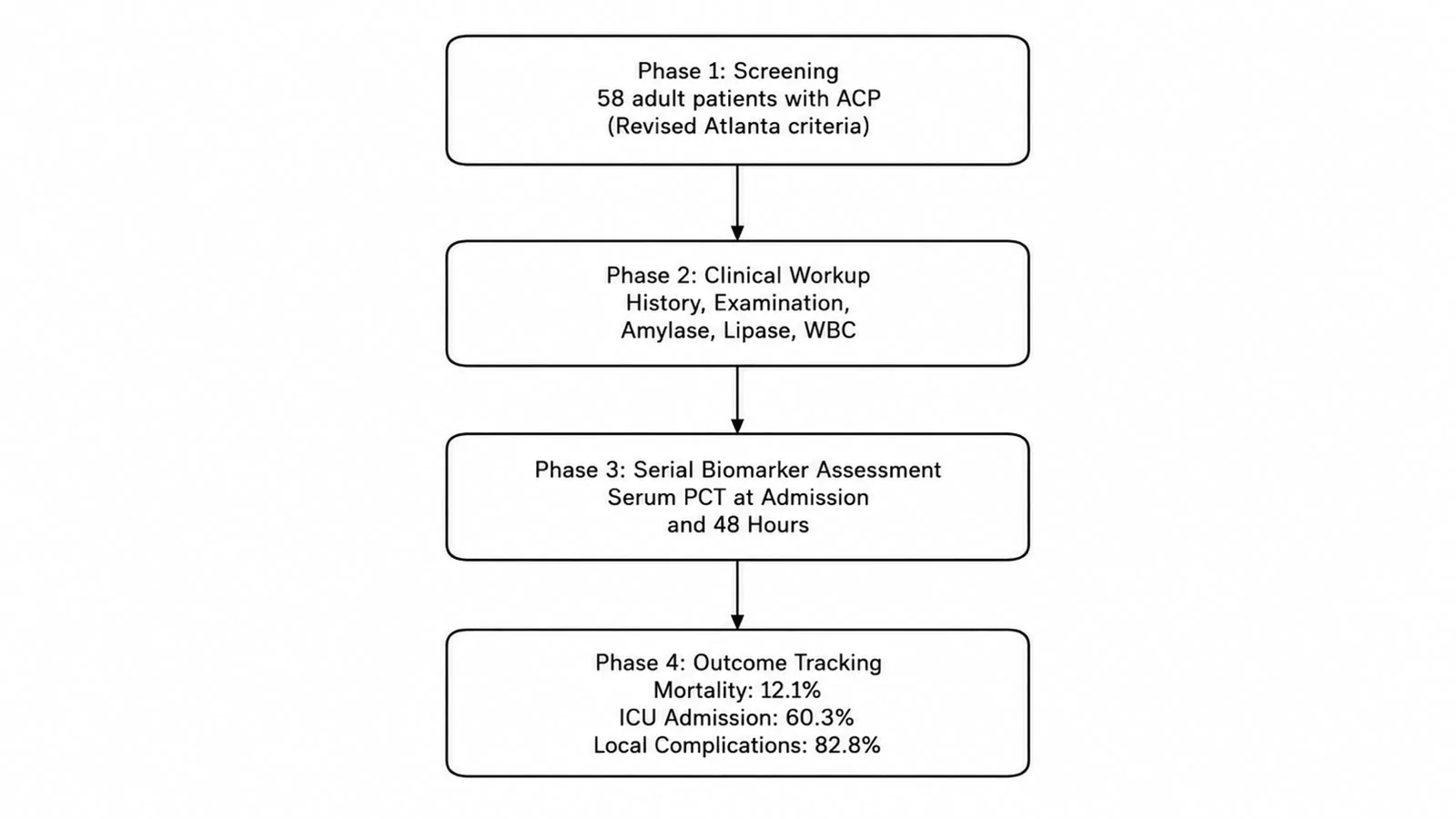
Study methodology ACP: acute-on-chronic pancreatitis; WBC: white blood cells; PCT: procalcitonin; ICU: intensive care unit

Data were analyzed using SPSS Statistics version 25.0 (IBM Corp., Armonk, NY). Continuous variables were tested for normality using the Shapiro-Wilk test and expressed as mean ± standard deviation (SD) or median with interquartile range (IQR), as appropriate. Categorical variables were presented as frequencies and percentages. Receiver operating characteristic (ROC) curve analysis was performed to evaluate the predictive accuracy of PCT for adverse outcomes, with area under the curve (AUC) values reported. Logistic regression models were constructed to identify independent predictors of mortality and organ failure, adjusting for confounders such as age, comorbidities, and alcohol use. A p-value < 0.05 was considered statistically significant. This rigorous statistical approach ensured robust evaluation of PCT’s prognostic utility in ACP. A schematic representation of the methodology is provided in Figure 1.

## Results

Cohort characteristics

A total of 58 adult patients with ACP were enrolled. The mean age was 43.9 years, with nearly half of the participants (n = 28; 48.28%) aged ≤ 40 years, indicating a substantial burden of disease among younger individuals. Males comprised 55.2% (n = 32) of the cohort, while females accounted for 44.8% (n = 26). Lifestyle analysis revealed alcohol consumption in 53.4% (n = 31) and smoking in 51.7% (n = 30), underscoring their prevalence as important background risk factors. The mean BMI was 25.03 ± 4.18 kg/m², placing the average participant in the overweight category, although values ranged from normal to obese. No significant correlation was observed between BMI and PCT levels. Patients were prospectively followed from emergency admission through serial 48-hour testing and assessment of final clinical outcomes at a tertiary care center (Table 1).

**Table 1:** Baseline characteristics of the study population (n = 58) SD: standard deviation

Parameter	Value
Age, years, mean ± SD	43.9 ± 17.0
Gender	
Male, n (%)	32 (55.2%)
Female, n (%)	26 (44.8%)
BMI, kg/m², mean ± SD	25.03 ± 4.18
Alcohol consumption, n (%)	31 (53.4%)
Pre-existing liver/kidney disease, n (%)	34 (58.6%)

Clinical presentation

All patients presented with abdominal pain radiating to the back, a hallmark feature of ACP. Vomiting was reported in 51.7% (n = 30), while loss of appetite affected 53.4% (n = 31). Nausea was less frequent, occurring in 32.8% (n = 19). Imaging revealed necrosis in 20.7% (n = 12) of patients exceeding 30% and in 19.0% (n = 11) with < 30% necrosis. Ultrasound and magnetic resonance cholangiopancreatography (MRCP) identified calcifications in 31% (n = 18) and ductal strictures in 25.9% (n = 15), consistent with chronic disease changes (Table 2). Overall, 82.8% (n = 48) developed at least one local complication, ranging from acute peripancreatic fluid collections to walled-off necrosis, reflecting the high disease burden.

**Table 2:** Clinical and laboratory findings at admission SD: standard deviation; CT: computed tomography; USG: ultrasonography

Parameter	Value
Abdominal pain, n (%)	58 (100%)
Vomiting, n (%)	30 (51.7%)
Serum amylase, U/L, mean ± SD	1,678.22 ± 752.56
Serum lipase, U/L, mean ± SD	2,312.19 ± 1,093.88
WBC count, /mm³, mean ± SD	11,378.43 ± 3,801.29
Serum calcium, mg/dL, mean ± SD	8.90 ± 0.93
Admission procalcitonin, ng/mL, mean ± SD	2.64 ± 1.47
48-hour procalcitonin, ng/mL, mean ± SD	2.48 ± 1.51
Pancreatic necrosis > 30% (CT), n (%)	12 (20.7%)
Pancreatic calcifications (USG), n (%)	18 (31.0%)

Baseline laboratory findings

Admission biochemistry demonstrated marked derangements. Mean serum amylase was 1678.22 ± 752.56 U/L, with only 24.1% (n = 14) of patients having values within normal limits. Lipase was similarly elevated (mean 2312.19 ± 1093.88 U/L). The mean WBC count was 11,378.43/mm³, with 72.4% (n = 42) showing leukocytosis. Admission PCT levels varied widely (mean 2.64 ± 1.47 ng/mL; range 0.14-4.99 ng/mL). Additional abnormalities included borderline hypocalcemia (mean 8.90 ± 0.93 mg/dL) and raised creatinine levels (mean 1.54 ± 0.53 mg/dL), indicating early metabolic and renal dysfunction in a subset of patients (Table 2).

Serial PCT trends

**Figure 2:**
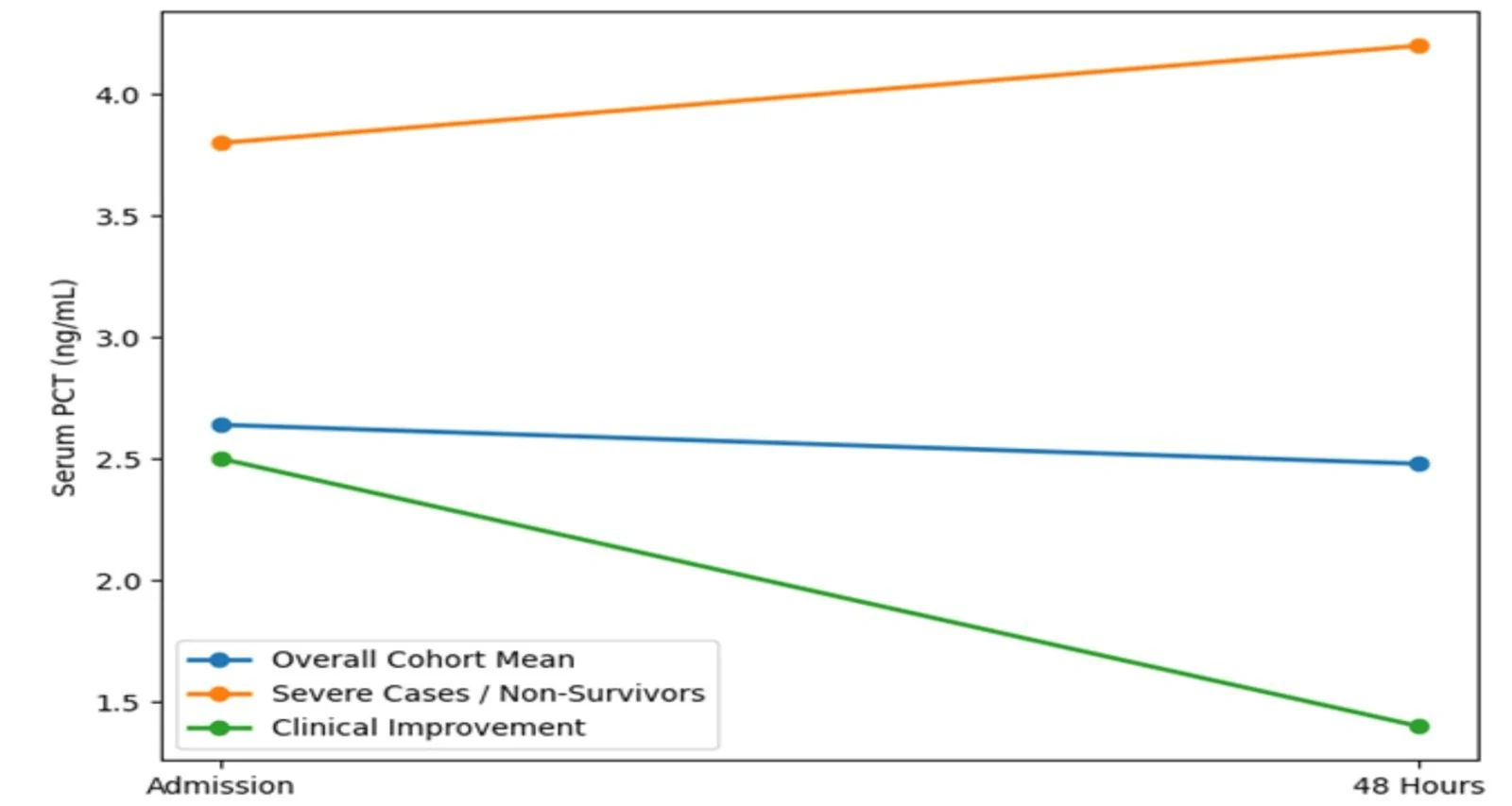
Serial procalcitonin trends

Dynamic changes in PCT provided critical prognostic insights. At admission, the mean PCT level was 2.64 ± 1.47 ng/mL. After 48 hours, the mean level declined slightly to 2.48 ± 1.51 ng/mL, although the overall values concealed divergent trajectories. Patients progressing to severe illness or death exhibited persistently elevated or rising PCT levels, whereas those who improved clinically showed a steady decline. This pattern demonstrated that serial PCT measurements, rather than a single baseline value, were more predictive of disease progression and complications (Figure 2).

Clinical outcomes

**Figure 3:**
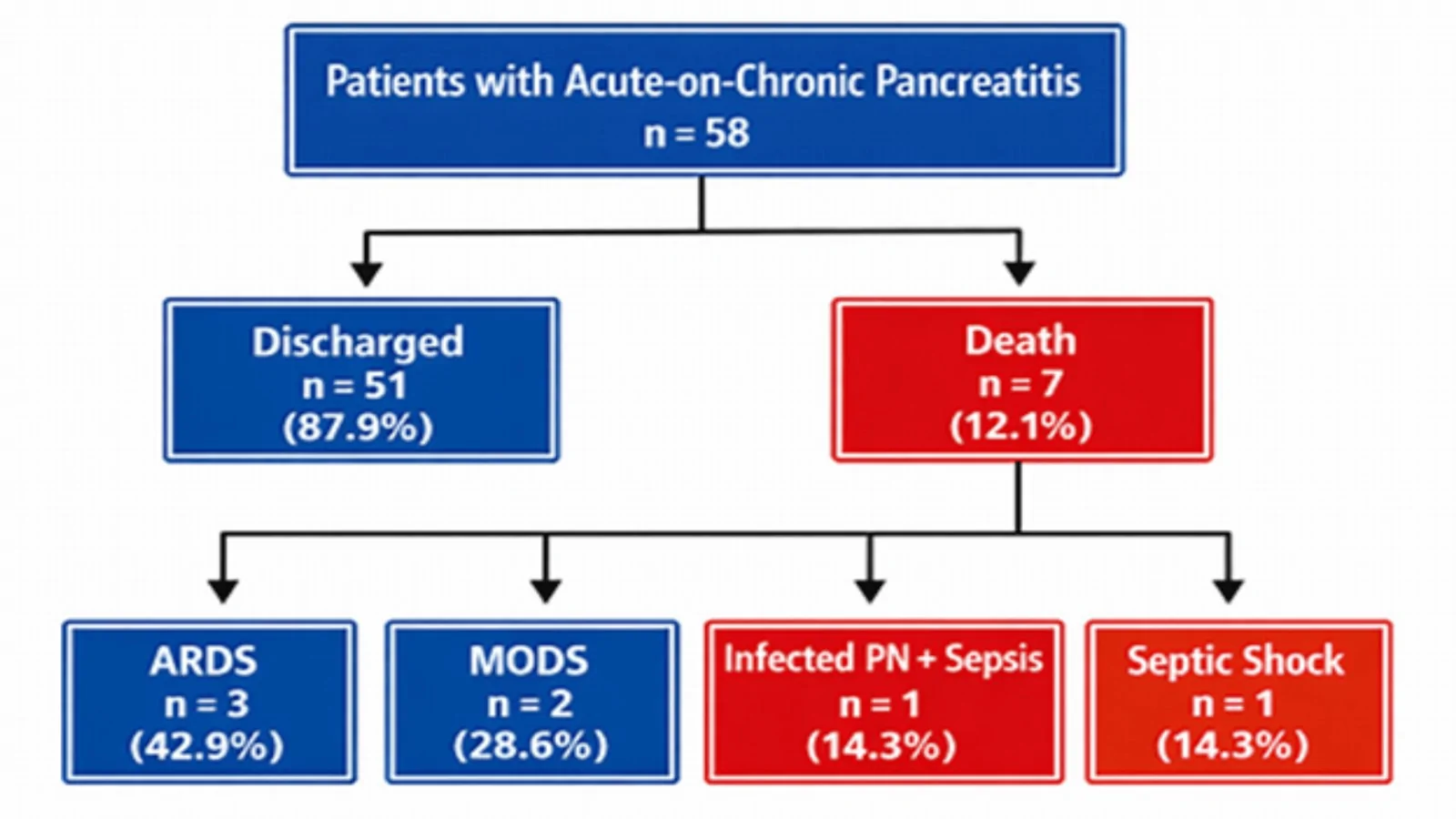
Clinical outcomes and causes of mortality in acute-on-chronic pancreatitis ARDS: acute respiratory distress syndrome; MODS: multiple organ dysfunction syndrome

Morbidity and mortality were considerable. Seven patients (12.1%; n = 7) died during hospitalization (Figure 3).

ICU admission was required in 60.3% (n = 35) of patients due to organ failure or systemic complications. Local complications were frequent (82.8%; n = 48), including peripancreatic fluid collections (22.4%; n = 13), necrosis (20.7%; n = 12), and walled-off necrosis (15.5%; n = 9). Length of stay varied widely, influenced by disease severity and comorbidities. Survivors accounted for 87.9% (n = 51) of patients at discharge, while patients with multiple organ failure (Modified Marshall Score > 3) had significantly worse outcomes (p = 0.0003), highlighting the complexity of ACP management (Table 3).

**Table 3:** Outcomes according to PCT levels PCT: procalcitonin; ICU: intensive care unit

Outcome	Normal PCT (n = 9), n (%)	Elevated PCT (n = 49), n (%)	P-value
Acute organ failure	2 (22.2%)	29 (59.2%)	0.021
Local complications	2 (22.2%)	32 (65.3%)	0.041
ICU admission	5 (55.6%)	30 (61.2%)	1.000
Mortality	0 (0.0%)	7 (14.29%)	0.79

Association between PCT and outcomes

**Figure 4:**
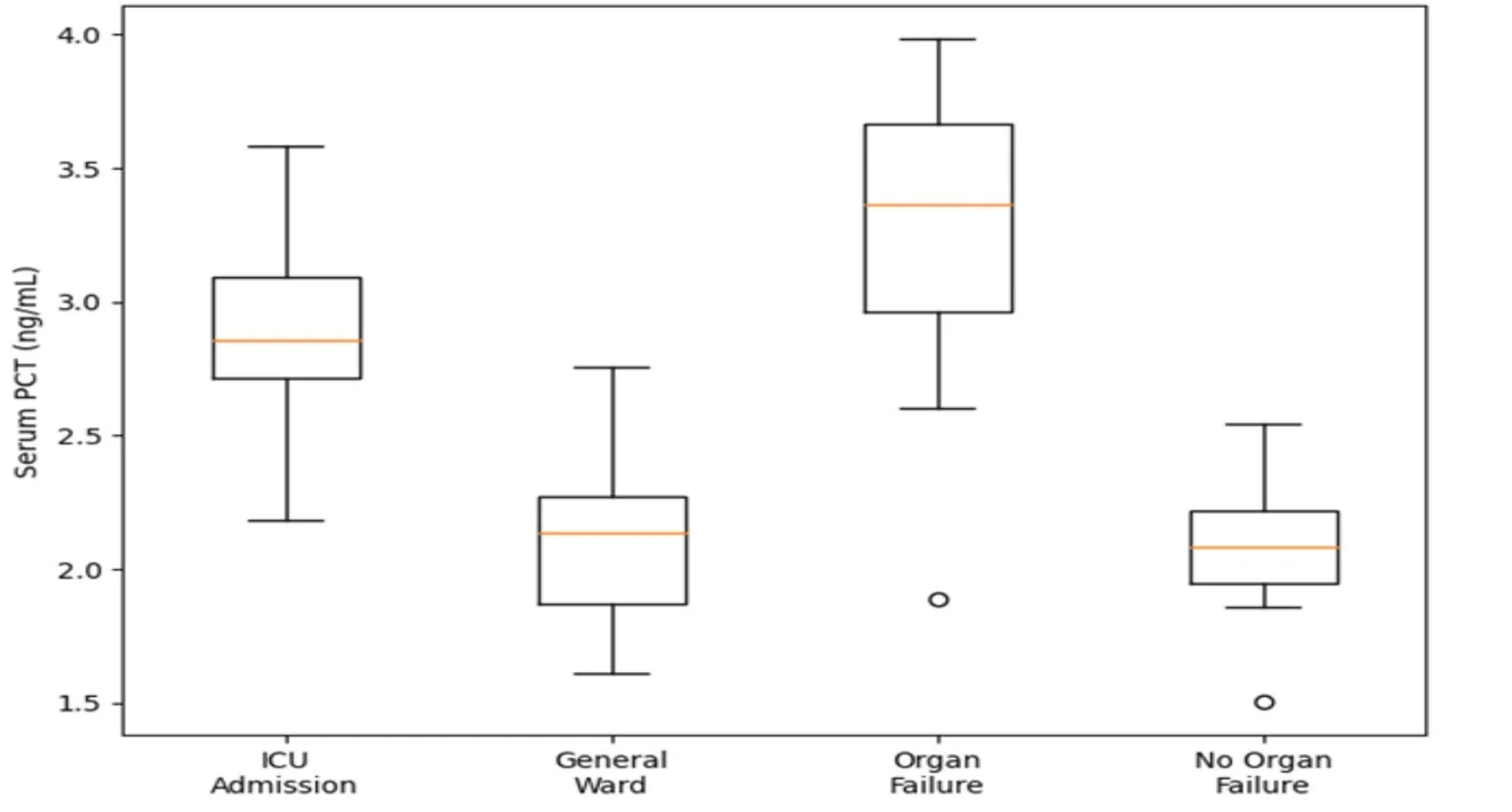
Association between PCT levels and clinical severity PCT: procalcitonin; ICU: intensive care unit

Higher PCT levels were strongly associated with adverse outcomes. Patients admitted to the ICU had significantly elevated mean PCT levels (2.95 ± 1.45 ng/mL) compared with those managed in the ward (2.20 ± 1.50 ng/mL; p = 0.034). Although admission PCT did not directly predict mortality, non-survivors consistently had higher values. Strong correlations were observed between PCT levels and length of hospital stay (r = 0.969 at admission; r = 0.979 at 48 hours; p = 0.0001). Elevated PCT was also associated with acute organ failure (p = 0.021) and local complications (p = 0.041), confirming its role as a marker of systemic inflammatory escalation (Figure 4).

ROC analysis

**Figure 5:**
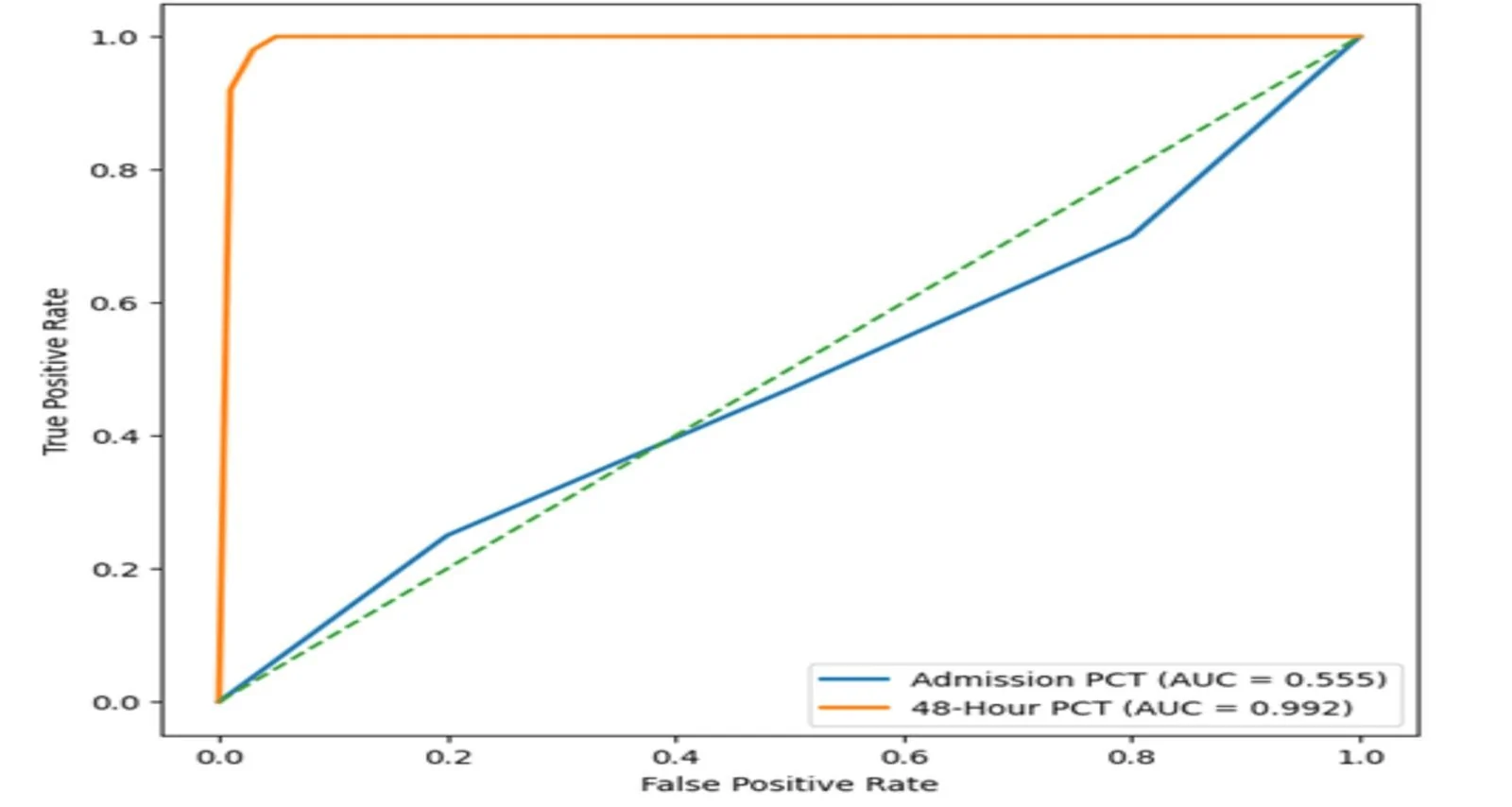
ROC curve ROC: receiver operating characteristic; PCT: procalcitonin; AUC: area under the curve

ROC analysis highlighted the superiority of 48-hour PCT over admission values. Admission PCT yielded poor discriminatory ability (AUC = 0.555; sensitivity = 46.9%; specificity = 76.9% at a cutoff of 3.46 ng/mL). In contrast, 48-hour PCT achieved excellent accuracy (AUC = 0.992). At a threshold of 4.04 ng/mL, sensitivity reached 100% with a negative predictive value (NPV) of 100%, specificity was 92.3%, and the good predictive value was 94.1%. These findings establish 48-hour PCT as a reliable predictor of mortality and severe morbidity (Table 4) (Figure 5).

**Table 4:** ROC analysis for predicting mortality ROC: receiver operating characteristic; AUC: area under the curve; PCT: procalcitonin; NPV: negative predictive value

Marker	AUC	Best threshold	Sensitivity	Specificity	NPV
Admission PCT	0.555	3.46 ng/mL	46.9%	76.9%	54.1%
48-Hour PCT	0.992	4.04 ng/mL	100%	92.3%	100%

Mortality

Among the seven patients who died during hospitalization, acute respiratory distress syndrome (ARDS) was the most common cause of mortality, accounting for three deaths (42.9%; n = 3). Multiple organ dysfunction syndrome (MODS) was responsible for two deaths (28.6%; n = 2). One patient died due to infected pancreatic necrosis complicated by sepsis, while another succumbed to septic shock. These findings indicate that mortality in ACP was primarily driven by severe systemic inflammatory and infectious complications, leading to organ dysfunction and failure.

**Table 5:** Causes of mortality among non-survivors PCT: procalcitonin; ARDS: acute respiratory distress syndrome; MODS: multiple organ dysfunction syndrome

Cause of death	N	%	Average PCT value (= 48 hrs)
ARDS	3	42.9	7.28
MODS	2	28.6	6.77
Infected pancreatic necrosis with sepsis	1	14.3	6.24
Septic shock	1	14.3	6.19
Total	7	100.0	

## Discussion

The present study demonstrates a substantial disease burden in ACP, characterized by a mortality rate of 12.1% and an ICU admission rate of 60.3% [3]. The most significant finding was the contrast in prognostic utility between the two time points. While admission PCT showed limited predictive value (AUC = 0.555), the 48-hour measurement demonstrated excellent accuracy (AUC = 0.992) for predicting mortality. Furthermore, serial PCT levels showed a very strong positive correlation with the duration of hospitalization (r > 0.95), confirming their utility as dynamic markers of severity. The superior performance of the 48-hour measurement reflects the kinetics of the inflammatory cascade. As the condition progresses, acinar cell damage triggers a systemic inflammatory response, during which multiple organs release PCT [10,17]. This process leads to cytokine amplification and delayed organ dysfunction, which becomes detectable within the 48-hour window [1,20]. Our findings mirror existing AP literature, in which Rau et al. [18] and Kylänpää-Bäck et al. [12] established PCT as a predictor of infected necrosis and organ failure, respectively.

In the unique context of ACP, it is hypothesized that pre-existing fibrosis may act as a protective barrier, potentially leading to a lower mortality risk compared to AP in a naïve pancreas [21,22]. This structural change also alters the biomarker profile; ACP patients often show lower-than-normal increases in pancreatic enzymes like amylase and lipase because chronic damage has caused significant tissue loss [6,23,24]. While enzymes lack prognostic utility, PCT reflects the true clinical complexity by matching the severity of SIRS activation and organ failure [17]. The clinical implications suggest that serial PCT monitoring should be integrated into triage protocols. In resource-limited settings, the 100% NPV at a threshold of 4.04 ng/mL provides a powerful tool for ruling out mortality risk and rationalizing ICU allocation [25]. Future research should validate these thresholds in larger multicentric cohorts to optimize risk stratification in this unique patient population.

The clinical implications of these findings are especially important in tertiary care and resource-limited healthcare settings, where early identification of high-risk patients is essential for appropriate allocation of intensive care resources and timely therapeutic intervention. Patients with persistently elevated or rising PCT levels demonstrated a significantly greater likelihood of developing organ dysfunction and local pancreatic complications, suggesting that serial PCT assessment may assist clinicians in identifying patients who require closer monitoring and aggressive supportive management. Conversely, lower 48-hour PCT levels demonstrated excellent negative predictive value, potentially allowing clinicians to identify lower-risk patients suitable for management in general wards or for earlier step-down care.

Compared with invasive diagnostic approaches such as fine-needle aspiration, serial PCT measurement provides a rapid, minimally invasive, and clinically practical alternative for prognostic assessment. Furthermore, the strong association between serial PCT dynamics and duration of hospital stay suggests that this biomarker may also assist healthcare systems in anticipating resource utilization and planning patient care more effectively. Incorporating serial PCT monitoring into routine clinical protocols may therefore improve individualized management strategies and optimize decision-making throughout the course of ACP.

The present study also demonstrated that PCT possesses several advantages over conventional inflammatory and pancreatic biomarkers in ACP. Although CRP is widely used to assess inflammatory severity, its slower kinetic response limits its utility during the critical early phase of disease progression. In contrast, the rapid rise of PCT allows earlier identification of patients at increased risk of severe complications. Traditional pancreatic enzymes such as amylase and lipase remain useful for establishing the diagnosis of pancreatitis; however, their prognostic utility is limited because serum levels often normalize regardless of disease severity.

In ACP specifically, chronic acinar destruction frequently leads to only modest enzyme elevation, thereby reducing their reliability as indicators of clinical progression. In the present cohort, admission amylase and lipase levels were elevated but did not independently predict adverse outcomes. PCT, on the other hand, appeared to more accurately reflect the magnitude of systemic inflammatory activation and organ dysfunction, thereby offering a broader assessment of disease trajectory than biomarkers confined primarily to pancreatic tissue injury. Furthermore, incidentally, the strong negative correlation found between vitamin D₃ levels and hospital stay (r = −0.832) suggested that nutritional status may further influence recovery [26]

Analysis of mortality patterns demonstrated that respiratory failure and systemic organ dysfunction were the predominant terminal events. ARDS accounted for nearly half of all deaths, highlighting the critical role of systemic inflammatory response and pulmonary complications in severe pancreatitis. The occurrence of MODS, septic shock, and infected pancreatic necrosis with sepsis further emphasizes that mortality in ACP is largely attributable to persistent organ failure and secondary infectious complications rather than localized pancreatic injury alone. These observations are consistent with existing literature indicating that organ failure remains the principal determinant of mortality in severe pancreatitis.

Several strengths of the study should be acknowledged. The prospective observational design enabled systematic collection of clinical, biochemical, and radiological data in a standardized manner. An additional methodological strength was the use of serial biomarker assessment rather than reliance on a single baseline value, allowing evaluation of dynamic inflammatory changes over time. Moreover, the study specifically focused on a relatively understudied but clinically important subgroup of patients with imaging-confirmed ACP, thereby providing disease-specific evidence that is frequently underrepresented in broader pancreatitis research.

However, certain limitations must also be considered. The study was conducted at a single tertiary care centre, which may limit generalizability to other healthcare settings and patient populations. The relatively small sample size also reduced statistical power, particularly for mortality-related subgroup analyses. In addition, the observational design precludes causal inference between elevated PCT levels and adverse clinical outcomes. The study did not directly compare PCT with established prognostic scoring systems such as APACHE II or BISAP, nor with emerging inflammatory biomarkers including interleukin-6. Furthermore, long-term follow-up beyond hospitalization was unavailable, preventing evaluation of recurrence rates, chronic disease progression, and long-term survival outcomes. Therefore, although the identified 48-hour PCT threshold demonstrated excellent predictive performance within this cohort, further validation in larger multicenter studies remains necessary before widespread clinical application can be recommended.

Future research should focus on validating serial PCT thresholds in larger multicentric cohorts involving diverse demographic and clinical populations. Combining serial PCT measurements with established severity scoring systems such as BISAP or Modified Marshall scores may improve predictive accuracy and facilitate development of more comprehensive prognostic models. Additional exploration of multimarker approaches incorporating inflammatory, metabolic, and nutritional parameters may further enhance risk stratification in ACP. Emerging applications of artificial intelligence and machine learning also offer promising opportunities for developing predictive algorithms capable of identifying high-risk patients early in the disease course. As ACP becomes increasingly recognized as a distinct clinicopathological entity, the development of a dedicated ACP-specific prognostic scoring system incorporating serial inflammatory kinetics and chronic pancreatic structural changes may substantially improve patient management and outcome prediction.

The present study demonstrates that serial serum PCT monitoring, particularly at 48 hours after admission, may serve as a highly effective prognostic tool in patients with ACP. While admission PCT values alone demonstrated limited predictive utility, serial assessment provided excellent discrimination for mortality and severe disease outcomes. These findings emphasize the importance of systemic inflammatory kinetics, rather than isolated pancreatic injury markers, in determining prognosis in ACP. Integration of serial PCT monitoring into structured management protocols may improve early risk stratification, optimize healthcare resource allocation, and support more individualized patient care in this complex inflammatory disorder.

## Conclusions

The present study demonstrates that serial serum procalcitonin monitoring, particularly at 48 hours after admission, may serve as a highly effective prognostic tool in patients with acute-on-chronic pancreatitis. While admission PCT values alone demonstrated limited predictive utility, serial assessment provided excellent discrimination for mortality and severe disease outcomes. These findings emphasize the importance of systemic inflammatory kinetics, rather than isolated pancreatic injury markers, in determining prognosis in ACP. Integration of serial procalcitonin monitoring into structured management protocols may improve early risk stratification, optimize healthcare resource allocation, and support more individualized patient care in this complex inflammatory disorder.
